# Fast and High-Resolution Imaging of Pollinated Stigmatic Cells by Tabletop Scanning Electron Microscopy

**DOI:** 10.21769/BioProtoc.5110

**Published:** 2024-11-20

**Authors:** Lucie Riglet, Isabelle Fobis-Loisy

**Affiliations:** 1 The Sainsbury Laboratory, University of Cambridge, 47 Bateman Street, Cambridge, UK; 2 Laboratoire Reproduction et Développement des Plantes, Univ Lyon, ENS de Lyon, UCB Lyon1, CNRS, INRAE, Lyon, France

**Keywords:** Scanning electron microscopy, Stigmatic cell, Pollen grain, Pollen tube path, Pollination, Hydration state

## Abstract

In plants, the first interaction between the pollen grain and the epidermal cells of the stigma is crucial for successful reproduction. When the pollen is accepted, it germinates, producing a tube that transports the two sperm cells to the ovules for fertilization. Confocal microscopy has been used to characterize the behavior of stigmatic cells post-pollination [1], but it is time-consuming since it requires the development of a range of fluorescent marker lines. Here, we propose a quick, high-resolution imaging protocol using tabletop scanning electron microscopy. This technique does not require prior sample fixation or fluorescent marker lines. It effectively captures pollen grain behavior from early hydration (a few minutes after pollination) to pollen tube growth within the stigma (1 h after pollination) and is particularly efficient for tracking pollen tube paths.

Key features

• Analysis of the pollen behavior in stigmatic cells of *Arabidopsis thaliana* but can be broadly used for other species.

• Rapid and high-resolution imaging method.

• Allows testing pollen grain hydration states, pollen tube paths on stigmatic cells from various genetic backgrounds, and also pollen tube phenotypes.

## Graphical overview


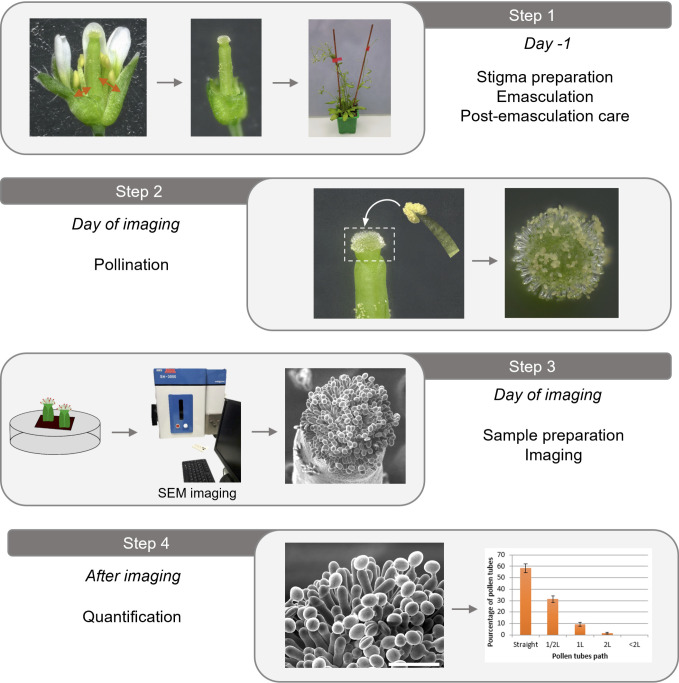


## Background

In plants, successful fertilization hinges on the early communication between the male pollen grain and the stigmatic epidermis of the female organ. The upper part of the pistil ends with a specialized tissue, the stigma, composed of hundreds of unicellular elongated epidermal cells, called stigmatic cells or papillae [2]. When a pollen grain lands on a papilla, it adheres and undergoes hydration. Subsequently, it germinates, producing a pollen tube that grows at the stigma surface and through the transmitting tract of the style and the ovary to transport male gametes toward the ovules for fertilization. Confocal microscopy has been used to visualize the early step of pollen–stigma interaction and better characterize the behavior of both partners post-pollination [1]. However, this method is time-consuming because it requires the production of fluorescent marker lines specific for both pollination partners. Furthermore, the commonly used ubiquitous promoters, such as the CAMV35S or ubiquitin 10 promoters, typically employed to drive the expression of fluorescent proteins in plant tissues, exhibit low activity in papillae and pollen. Therefore, it is required to utilize specific promoters to conduct the expression of fusion proteins in transgenic stigma and pollen.

Here, we share a rapid, high-resolution imaging protocol using tabletop scanning electron microscopy, used in Riglet et al. [3], that does not require prior sample fixation or fluorescent marker lines. It enables effective visualization of pollen–stigma interaction and characterization of the pollen behavior, capturing events from early hydration of the pollen grain (a few minutes after pollination) to pollen tube growth within the stigma (1 h after pollination). This method proves particularly efficient for assessing hydration states and tracking pollen tube paths; it is particularly suitable to compare the pollen tube path on a wild-type stigma with mutated stigmatic cells but can also be used in reverse, comparing mutated pollen grains and tube paths on wild-type stigmatic cells.

## Materials and reagents

**Biological materials**

1. *Arabidopsis thaliana*, ecotype Columbia Col-0 plants with flowers at stages 12–15 [4] in individual pots

**Other materials**

1. Straight fine tweezers (Dumont, model: Inox 8)
2. Double-sided black tape (Synergie4, model: DEN-77816)
3. Wood sticks and soft plant ties to stack the plants
4. Sewing thread to tag the emasculated pistils

## Equipment

1. Tabletop scanning electron microscope (Hirox, model: SH-3000)
2. Stereomicroscope (Nikon, model: SMZ645, total magnification from 8× to 50×; eyepieces 10)

## Software and datasets

1. ImageJ software (1.54c, 2024)]

## Procedure

- **Plant preparation**
  1. A few *Arabidopsis thaliana* seeds, accession Col0, were germinated in 0.25 L plastic pots containing a peat-based compost with 60 kg/m^3^ of clay. After one week, we remove plantlets to keep one plant per pot to facilitate manipulation during emasculation and pollination. Plants are maintained in a growth chamber under long-day conditions (16 h light, 120–150 µmol m^-2^·s^-1^/8 h dark at 21 °C/19 °C) with a relative humidity of around 60%. The plants are watered throughout their growth cycle. To prevent pest attacks, it is important to avoid overwatering, especially when the plants are young. Fertilizer (N/P/K: 18/10/18) is added to the watering water (4 mL/L) once a week.
  2. In our growth conditions, plants are ready for emasculation four weeks after germination; they have three or four robust stems. Avoid using old plants, which have more fragile stems and flowers and are more prone to dehydration during emasculation.
  3. Water the plant thoroughly the day before emasculation to ensure turgid stigmas and pollen.
  4. Flower emasculation should be done the day before pollination and imaging. Perform all processes at room temperature (around 22 °C).
- **Stigma preparation (emasculation procedure, the day before)**
  Flower emasculation should be done the day before pollination and imaging. See Video 1.
  1. Position the plant in its culture pot under a stereomicroscope.
  2. Bend the plant's inflorescences and gently clamp a flower bud at the end of stage 12 (Figure 1, Smyth et al. [4]) between the thumb and the forefinger to prevent bud movement during emasculation.
    **Video 1.**
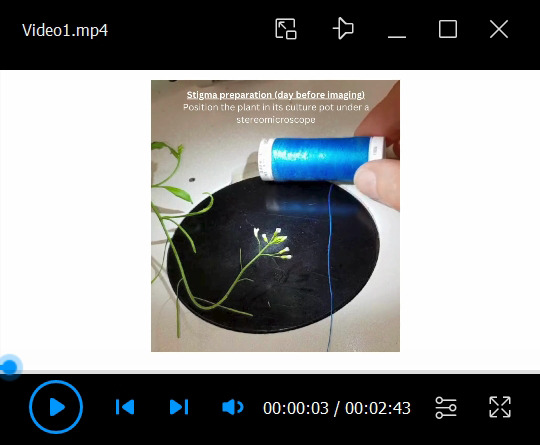
Emasculation and pollination procedures
    **Figure 1.**
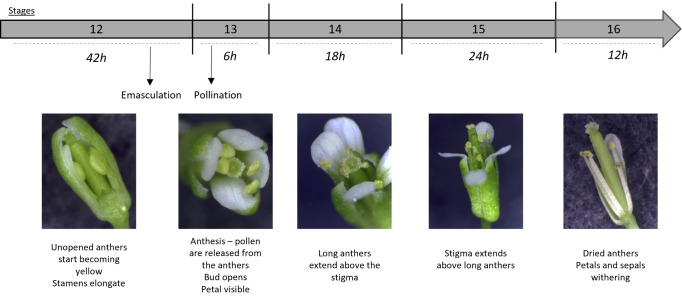
*Arabidopsis thaliana* developmental stages, focused on the stigma and pollen maturity. Inspired from Smyth et al. [4]. Stage duration is specified. A sepal from a stage-12 flower bud was removed to better visualize internal organs.
  3. Using straight fine tweezers, remove flowers older than stage 12 [4] to minimize the risk of cross-pollination and younger buds to facilitate access to the stage-12 bud.
  4. Using fine tweezers, carefully open the flower bud to expose the stigma (Figure 2A, B). Verify that anthers are not dehiscent, without any pollen grain released.
*Note: Emasculate in the morning when the anthers are less dehiscent, typically when the growth chamber lights are just starting, to minimize the risk of pollen contamination from other stigmas.*
  5. Carefully remove the anthers (pale yellow, Figure 2C), petals, and sepals.
*Note: Instead of completely removing organs, cut them with the fine tweezers (as indicated by arrows in Figure 2C) to retain part of the tissues (Figure 2D) and prevent excess dehydration of the bare pistil.*
  6. Ensure that no pollen grains remain on the stigma papillae (Figure 2E).
**Caution:** If any pollen grains are present, discard the bud and select another stage-12 bud.
*Note: Limit emasculation to no more than five buds per plant to prevent pistil dehydration or damage to the emasculated buds while manipulating the plant.*
    **Figure 2.**
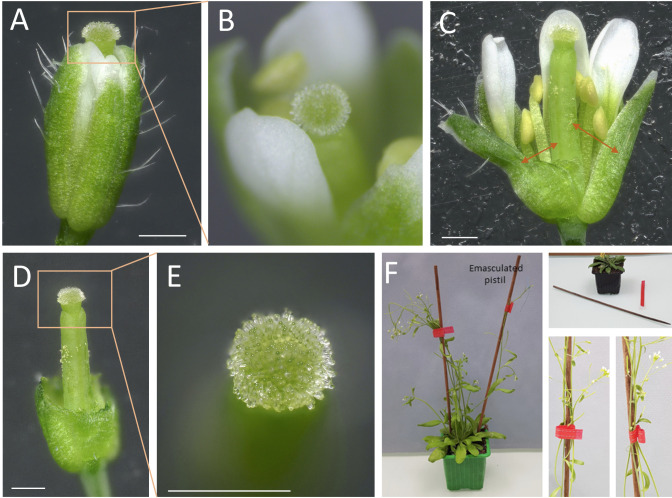
*Arabidopsis thaliana* stigma at the end of stage 12 and emasculation prior to pollination. (A) Overview of the entire flower bud at the end of stage 12 with sepals and petals pushed back to better visualize the pistil. Orange arrows indicating the site for emasculation. (B) Close-up of the upper part of the flower. (C) Flower (manually opened) with orange arrows indicating the site for emasculation by anther removal. (D) Complete view of the emasculated flower. (E) Detailed close-up of the unpollinated stigma, free of pollen grains. Scale bars, 500 μm. (F) Staking of the plants using wood sticks and soft plant ties to support their stems.
- **Post-emasculation care**
  1. To label the emasculated pistil, a sewing thread can be attached at the base of the bare pistil.
  2. Stake the plants (Figure 2F). We use wood sticks and soft plant ties to support their stems (see Graphical overview, step 1).
    Caution: This step is important to prevent the bare pistils from getting in contact with pollen from other flower buds.
  3. Return the plants to the culture room. If imaging stage-13 stigmas, wait approximately 18 h after emasculation (Figure 1; Smyth et al. [4]).
*Note: Ensure the plant is well-watered to maintain turgescence in the stigma and pollen.*
- **Pollination procedure (the day of imaging)**
  Under a stereomicroscope, hand-pollinate each emasculated flower with mature wild-type pollen grains (e.g., flowers at stage 13, Figure 1 and 3A).
  1. To do so, collect a single dehiscent anther from a non-emasculated flower bud with tweezers and carefully brush the stigma with it. For easier quantification of pollen tube paths, deposit only a small amount of pollen grains (as shown in Figure 3B, C). To achieve this, gently brush the dehiscent anther on a tissue to remove excess pollen grains before applying it to the stigma.
*Note: Collect a dehiscent anther from stage 13 to early stage 14 with pollen grains not too old to ensure the highest germination rate.*
*Note on pollen germination: Temperature and humidity influence pollen germination. Therefore, prepare and store samples in a room at a moderate temperature (around 20 °C) and ensure it is not too dry (at least 50% humidity). This environment helps maintain optimal conditions for pollen germination.*
    **Figure 3.**
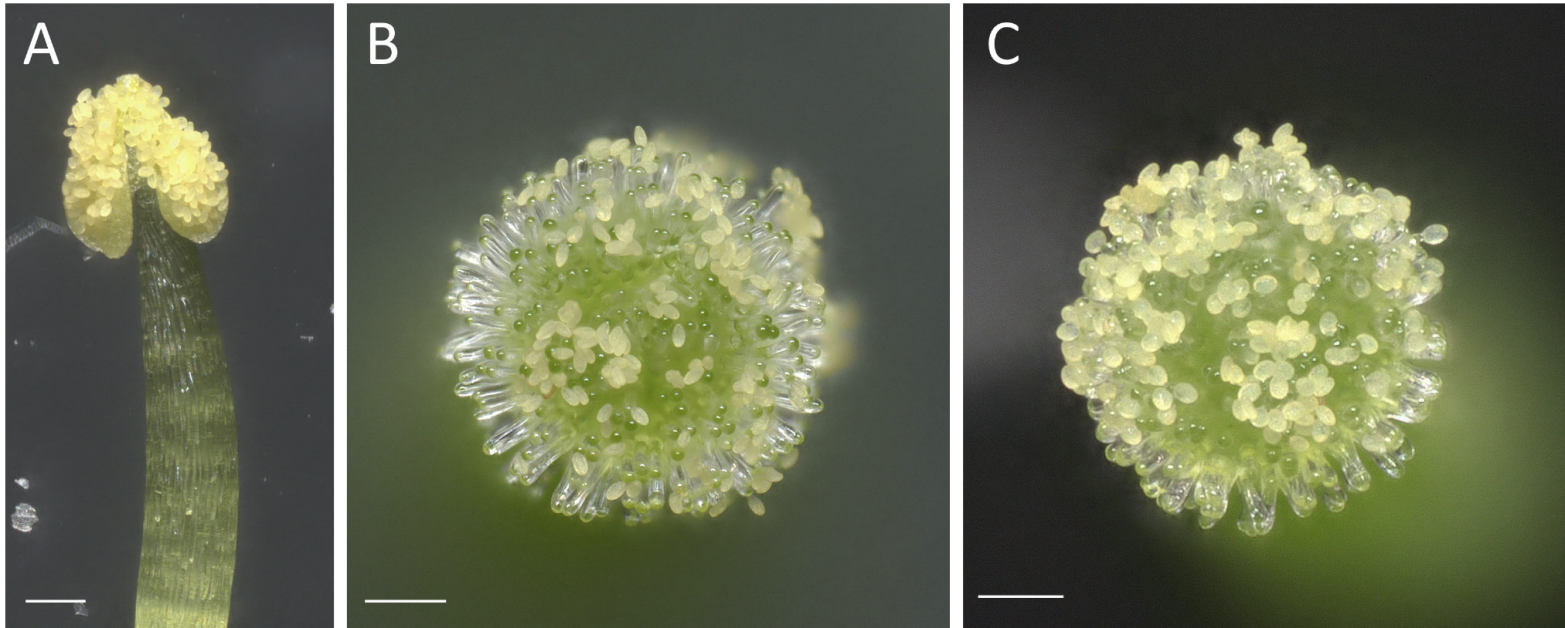
Pollination of *Arabidopsis thaliana* stigma at stage 13, stereomicroscope view. (A) Anther at maturity a few hours after dehiscence with yellow pollen grains. (B) Stigma shortly after pollination, displaying non-hydrated oval pollen grains (rugby balloon–like shape). (C) Stigma 30 min post-pollination, exhibiting well-hydrated roundish pollen grains (basket balloon–like shape). Scale bars, 100 μm.
  2. Post-pollination handling: Leave the pollinated plants at room temperature on the bench. Depending on the focus of the imaging:
    - Wait 10–15 min to image pollen hydration.
    - Wait 30–60 min to image long pollen tubes.
- **Sample preparation**
  1. Cut the pollinated pistils transversally in the middle of the ovary using fine tweezers at the designated time post-pollination (see Figure 4).
**Caution:** Ensure that the sample height does not exceed 0.5 cm to fit into the microscope chamber.
    **Figure 4.**
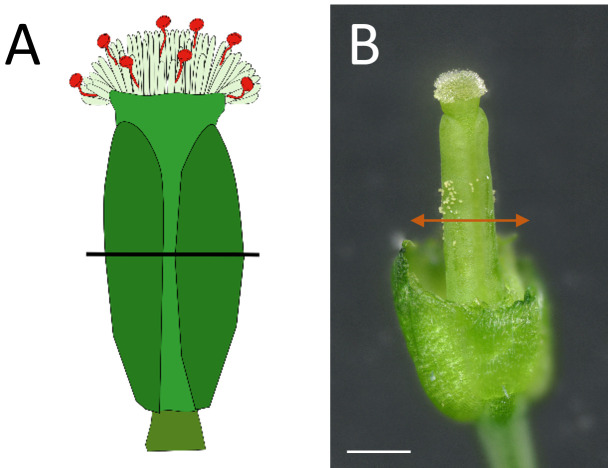
Preparation of the sample before deposition on the scanning electron microscopy platform. (A–B) Position of the transversal cut of the pistil in the middle of the ovary.
- **Image setup procedure**
  1. Sample mounting: Gently deposit two or three cut pistils vertically onto a 4 × 2 cm double-sided tape positioned on the SEM platform (see Figure 5). Ensure that the pistils are not too close to each other, maintaining a minimum distance of 1 cm between both.
    **Figure 5.**
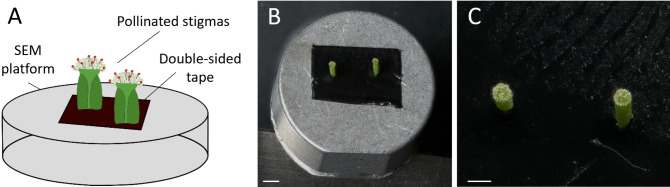
Scanning electron microscopy platform preparation. (A) Schematic view showing the method of mounting the stigmas on the SEM platform. (B) Platform displaying the cut pollinated pistils stuck on the double-sided tape before imaging. Scale bar, 1,000 μm. (C) Close-up view of two pistils mounted on the platform. Scale bar, 500 μm.
  2. Scanning electron microscopy setup: Place the platform in the Hirox SEM SH-3000 (Figure 6), ensuring the door of the SEM is well sealed.
*Note: The same protocol can be used with other tabletop SEMs.*
    **Figure 6.**
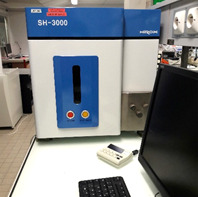
Scanning electron microscopy Hirox SH-3000
  3. Select temperature (-20 °C) and wait until that temperature is reached.
  4. Select accelerating voltage: 15 kV.
  5. Turn on the vacuum.
- **Imaging procedure**
  1. Observation: Observe samples within the first 15 min to prevent cells from collapsing during imaging.
**Caution:** Fast action is crucial as cells can collapse quickly within 15 min (see Figure 7A), making quantification of the pollen tube path impossible.
    For a quick initial screening, use the AutoFocus button.
*Tip: To obtain a sharp image of the stigma surface and adjust the focus, initially zoom in at high magnification on pollen grains located in the center of the stigma. Adjust the focus, contrast, and brightness. Then, zoom out to capture a broad view of the pistil.*
  2. Image capture: Acquire the images (Figure 7A–E). This method allows for imaging of the pollen hydration stage. Figure 7B displays non-hydrated pollen grains, while Figure 7C presents well-hydrated ones (Figure 7C). Pollen tube germination and tube paths can also be imaged and later quantified (Figure 7D–E) (See Data analysis section).
    **Figure 7.**
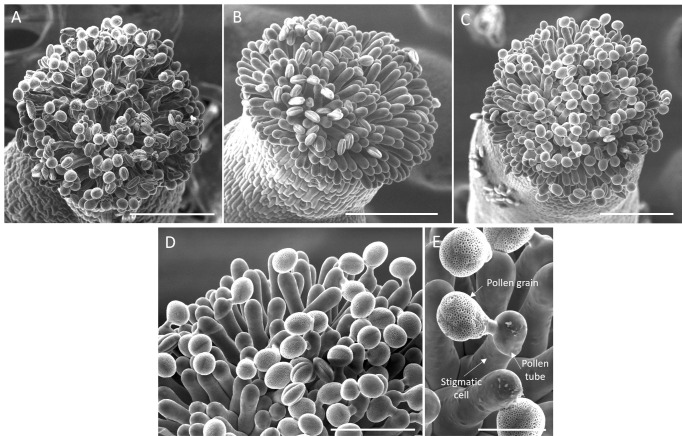
Visualization of pollinated stigmas at the scanning electron microscope. (A–C) Top view of a pollinated stigma observed at 300× magnification, showing (A) deflated stigmatic cells (collapsed), (B) fully turgid stigmatic cells with non-hydrated pollen grains (oval) a few minutes after pollination, and (C) stigmatic cells with well-hydrated pollen grains (round) and pollen tubes growing 30 min after pollination. (A–C) Scale bar, 50 μm. (D) Detail of (C) at 700× magnification. Scale bar, 50 μm. (E) Pollen–stigmatic cell interaction, viewed at 700× magnification. Scale bar, 20 μm.
  3. This method can be applied to compare the pollen tube paths on wild-type and mutated stigmatic cells. Additionally, it can be used reversely, comparing wild-type and mutated pollen grain hydration and pollen tube paths on wild-type stigmatic cells. To compare the behavior associated with a wild type and a mutant, ensure that the stigmatic cells are pollinated at the same development stage and that pollination is done simultaneously. Ideally, mount a wild type and a mutant on the same platform to ensure imaging is performed under identical conditions.
  4. Alternatively, single-stigma pollination can be conducted by applying two different pollen genotypes on each half of the stigma, as previously described [5].

## Data analysis

- **Data processing and analysis: Quantification of pollen tube turn numbers using ImageJ software**
  1. Image processing: Open the images in ImageJ software and use the *Cell Counter* plugin to record the number of turns made by pollen tubes on the stigmatic epidermis.
  2. Turn quantification: Count the number of turns made by the pollen tubes up to the base of the stigmatic cell.
*Notes:*
    - *Select only papillae that received a single pollen grain to facilitate tracking of the pollen tube path. Indeed, if multiple pollen tubes are growing on the same papilla, their path may be affected by another one, deviating from the initial direction. This property, which we defined as self-avoidance, is illustrated in Figure 8A [6].*
    - *Select papillae for which the entire cell is visible from the top to the base.*
  3. Direction categorization: Use the *Cell Counter* tool in ImageJ to categorize the direction taken by the pollen tube. Navigate through *Plugins* → *Analyze* → *Cell Counter*. Classify the turns into several categories (Figure 8B):
    - Type 1: Straight—the pollen grain germinates on one side of the papilla and the tube stays on the same side.
    - Type 2: Half a loop—the pollen grain germinates on one side of the papilla and the tube grows toward the other side.
    - Type 3: One full loop—the pollen grain germinates on one side of the papilla and the tube returns to the same side before reaching the stigmatic base.
    - Type 4: Two loops.
    - Type 5: More than two loops.
    An example of calculation and analysis is presented in Figure 8C, D.
    **Figure 8.**
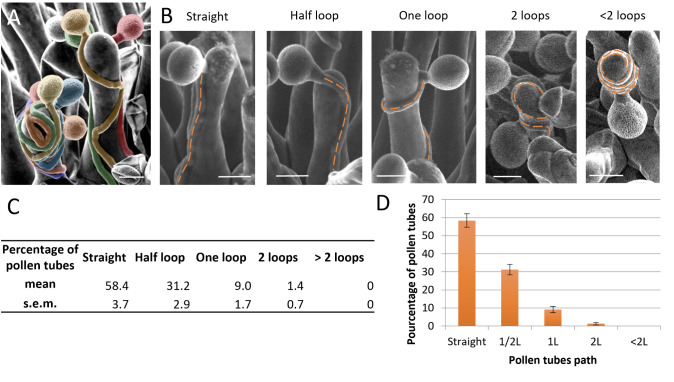
Pollen tube paths on *Arabidopsis thaliana* stigmatic cells. (A) SEM images of a wild-type stigmatic cell pollinated by two wild-type pollen grains illustrating self-avoidance properties. Each pollen grain and tube have been colored to better visualize their paths. (B) Different configurations of pollen tube paths on *Arabidopsis thaliana* stigmas. (C) Example of the quantification of the number of loops made by the pollen tube on papillae at stage 13 (data from Riglet et al. [3]). Data are expressed as mean ± standard error of the mean (SEM). (D) Example of a histogram illustrating the quantification of the number of loops formed by the pollen tube on papillae at stage 13 (data from Riglet et al. [3]).
- **Statistical analysis**
  1. At least three independent experiments (different dates and plant batches) have to be performed, with at least 50 pollinated papillae analyzed in each experiment.
  2. Data are expressed as mean ± SEM.
  3. Use an adjusted chi-square test for homogeneity to analyze differences between two genotypes. Employ a chi-square test for independence to compare the same genotype at different stages.

## Validation of protocol

This protocol or parts of it has been used and validated in the following research article(s):

- Riglet et al. [3]. KATANIN-dependent mechanical properties of the stigmatic cell wall mediate the pollen tube path in *Arabidopsis. eLife* (Figure 2 panels A, B; Figure 4 panels B, C; Figure 5 panels B, D; Figure 5, supplement 1 panels A–D; Figure 5, supplement 2 panel C, Figure 5, supplement 3 panels A–G).

## References

[1] Rozier F., Riglet L., Kodera C., Bayle V., Durand E., Schnabel J., Gaude T. and Fobis-Loisy I. (2020). Live-cell imaging of early events following pollen perception in self-incompatible *Arabidopsis thaliana* . J Exp Bot. 71(9): 2513-2526. 31943064 10.1093/jxb/eraa008 PMC7210763

[2] Heslop-Harrison Y. and Shivanna K. R. (1977). The Receptive Surface of the Angiosperm Stigma. Ann Bot. 41(6): 1233-1258.

[3] Riglet L., Rozier F., Kodera C., Bovio S., Sechet J., Fobis-Loisy I. and Gaude T. (2020). KATANIN-dependent mechanical properties of the stigmatic cell wall mediate the pollen tube path in *Arabidopsis* . eLife. 9: e57282. 32867920 10.7554/eLife.57282 PMC7462616

[4] Smyth D. R., Bowman J. L. and Meyerowitz E. M. (1990). Early flower development in *Arabidopsis* . Plant Cell. 2(8): 755-767. 2152125 10.1105/tpc.2.8.755 PMC159928

[5] Nasrallah M. E., Liu P. and Nasrallah J. (2002). Generation of Self-Incompatible *Arabidopsis thaliana* by Transfer of Two *S* Locus Genes from *A. lyrata* . Science. 297(5579): 247-249. 12114625 10.1126/science.1072205

[6] Riglet L., Quilliet C., Godin C., John K. and Fobis-Loisy I. (2024). Geometry and cell wall mechanics guide early pollen tube growth in *Arabidopsis thaliana* . bioRxiv. doi.org/ 10.1101/2024.02 .05.578915.

